# HLA Evolutionary Divergence as a Prognostic Marker for AML Patients Undergoing Allogeneic Stem Cell Transplantation

**DOI:** 10.3390/cancers12071835

**Published:** 2020-07-08

**Authors:** Malte Roerden, Annika Nelde, Jonas S. Heitmann, Reinhild Klein, Hans-Georg Rammensee, Wolfgang A. Bethge, Juliane S. Walz

**Affiliations:** 1 Department of Hematology, Oncology, Clinical Immunology and Rheumatology, University Hospital Tübingen, 72076 Tübingen, Germany; reinhild.klein@med.uni-tuebingen.de (R.K.); wolfgang.bethge@med.uni-tuebingen.de (W.A.B.); 2 Cluster of Excellence iFIT (EXC2180) “Image-Guided and Functionally Instructed Tumor Therapies”, University of Tübingen, 72076 Tübingen, Germany; annika.nelde@uni-tuebingen.de (A.N.); Jonas.Heitmann@med.uni-tuebingen.de (J.S.H.); 3 Clinical Collaboration Unit Translational Immunology, German Cancer Consortium (DKTK), Department of Internal Medicine, University Hospital Tübingen, 72076 Tübingen, Germany; 4 Institute for Cell Biology, Department of Immunology, University of Tübingen, 72076 Tübingen, Germany; rammensee@uni-tuebingen.de; 5 German Cancer Consortium (DKTK), DKFZ Partner Site Tübingen, 72076 Tübingen, Germany

**Keywords:** HLA evolutionary divergence, acute myeloid leukemia, AML, allogeneic stem cell transplantation, graft-versus-leukemia effect

## Abstract

The diversity of human leukocyte antigens (HLAs) enables the presentation of immense repertoires of peptides, including tumor-associated antigens (TAAs). As a surrogate for immunopeptidome diversity, the HLA evolutionary divergence (HED) between individual HLA alleles might directly define the ability to present TAAs, a prerequisite for graft-versus-leukemia effects. We therefore analyzed the impact of HED on survival within a cohort of 171 acute myeloid leukemia (AML) patients after matched donor allogeneic hematopoietic stem cell transplantation (HSCT). Low HED (<25th percentile) of HLA class I (HED^class I^) or HLA-DR antigens (HED^DR^) was a strong determinant for adverse overall survival after allogeneic HSCT (OS), with a hazard ratio for death of 1.9 (95% CI 1.2–3.2) and 2.1 (95% CI 1.3–3.4), respectively. Defining a cutoff value for the combined HED^total^ (HED^class I^ and HED^DR^), the respective 5 year OS was 29.7% and 64.9% in patients with low and high HED^total^ (*p* < 0.001), respectively. Furthermore, the risk of relapse was significantly higher in patients with low HED^total^ (hazard ratio (HR) 2.2, 95% CI 1.3–3.6) and event-free survival (EFS) was significantly reduced (5 year EFS 25.7% versus 54.4%, *p* < 0.001). We here introduce HED, a fundamental metric of immunopeptidome diversity, as a novel prognostic factor for AML patients undergoing allogeneic HSCT.

## 1. Introduction

Graft-versus-leukemia (GvL) effects and the success of allogeneic hematopoietic stem cell transplantation (HSCT) are based on the recognition of tumor-associated antigens (TAAs) presented to T cells via human leukocyte antigens (HLAs) [1,2,3]. HLAs are highly polymorphic and therefore each allele presents a distinct repertoire of peptides [4,5,6], collectively referred to as the immunopeptidome. A high HLA evolutionary divergence (HED), between the HLA alleles of an individual, might allow for the presentation of a more diverse immunopeptidome, and thus directly define the ability to present TAAs, a prerequisite for anti-cancer immune responses, including GvL effects [5,7,8,9]. The Grantham distance allows for the quantification of divergence between HLA alleles, taking into account the physiochemical differences of the respective binding-domain peptide sequences [4,10]. A recent study highlighted the significance of HED for cancer immunotherapy by demonstrating that a high HED is associated with immunopeptidome diversity and a superior outcome in patients undergoing immune checkpoint blockade therapy for solid malignancies [7]. The immunogenicity of acute myeloid leukemia (AML) is well established and allogeneic HSCT can achieve long-term disease control in a subset of acute myeloid leukemia patients. However, the factors governing GvL effects are incompletely understood and predicting survival after allogeneic HSCT remains difficult [11,12,13]. We therefore evaluated the significance of HED for the therapeutic outcome after allogeneic HSCT by retrospectively analyzing an AML patient cohort undergoing HLA-matched allogeneic HSCT.

## 2. Results

**Figure 1:**
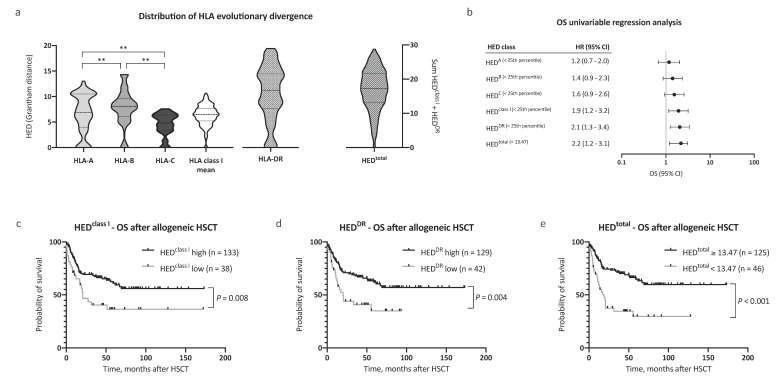
Distribution of human leukocyte antigen (HLA) evolutionary divergence and overall survival after allogeneic hematopoietic stem cell transplantation (HSCT). (**a**) Distribution of HLA evolutionary divergence (HED) with regard to HLA-A (25th percentile 3.99, median 6.89), HLA-B (25th percentile 6.11, median 8.05), HLA-C (25th percentile 3.39, median 4.82), HLA class I mean (25th percentile 5.23, median 6.50), and HLA-DR antigens (25th percentile 7.58, median 11.17), as well as the sum of HLA class I mean and HLA-DR (*n* = 242). (**b**) Univariable Cox regression analysis of the impact of low HED on overall survival (OS). (**c**–**e**) Kaplan–Meier analysis of OS with regard to HED of (**c**) HLA class I, (**d**) HLA-DR, and (**e**) HED^total^. Abbreviations: CI indicates confidence interval; HED, HLA evolutionary divergence; HSCT, hematopoietic stem cell transplantation; OS, overall survival; and **, *p <* 0.01 (Mann–Whitney test).

Firstly, pairwise divergences of HLA class I and HLA-DR alleles for all patients (*n* = 242) were calculated. In line with previous reports [7], we noted significant differences in pairwise divergence between HLA-A, -B, and -C alleles, with HLA-B alleles contributing most to mean HED^class I^ (median 6.89, 8.05, and 4.82, respectively, Figure 1a). Significantly, HLA-DR antigens showed the highest divergence among all analyzed HLA antigens (HED^DR^, median 11.17). Assuming equal contributions to antigen presentation, mean HED of HLA class I alleles (HED^class I^) and HED^DR^ were subsequently used for survival analysis.

Next, we evaluated the impact of HED^class I^ and HED^DR^ on survival. Patient characteristics were similar between compared groups with regard to age, performance status, disease risk scores (European LeukemiaNet (ELN) [14,15] and European Society for Blood and Marrow Transplantation (EBMT) score [11]), allogeneic HSCT setting, and remission status prior to HSCT, allowing for a comparison of outcomes (patient characteristics in Table 1). The mean age at allogeneic HSCT was 54.2 years, and the median follow-up time after allogeneic HSCT was 59.4 months. Low HED^class I^ and HED^DR^ were associated with a significantly reduced overall survival (OS) after allogeneic HSCT (hazard ratio (HR) 1.9, 95% CI 1.2–3.2, and HR 2.1, 95% CI 1.3–3.4, respectively, Figure 1b). The 5 year OS after allogeneic HSCT was 36.5% and 61.0% for patients in the low and high HED^class I^ cohort, respectively (Figure 1c, *p* = 0.008). In patients with low HED^DR^, the 5 year OS after allogeneic HSCT was 35.1% compared with 62.0% in patients with high HED^DR^ (Figure 1d, *p* = 0.004).

**Figure 2:**
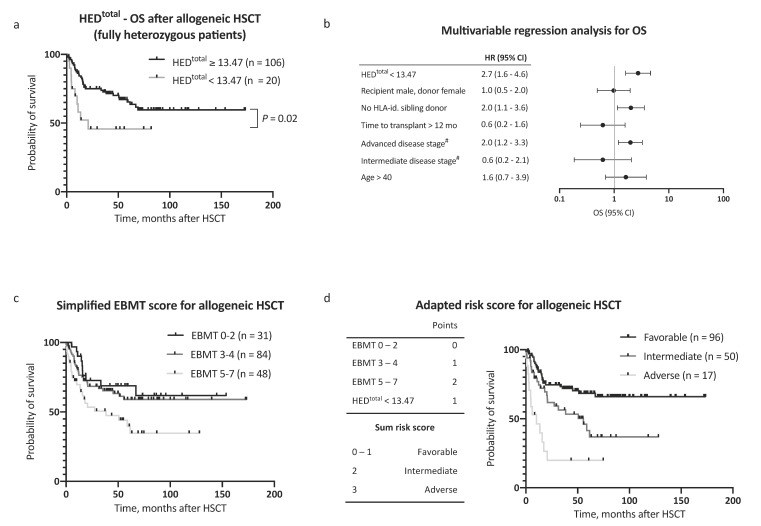
Overall survival multivariable regression analysis and risk score assessment. (**a**) Kaplan–Meier analysis of OS with regard to HED^total^, considering only patients fully heterozygous for HLA-A, HLA-B, HLA-C, and HLA-DR (*n* = 126). (**b**) Multivariable Cox regression analysis of the impact of low HED^total^ on OS. (**c**) Kaplan–Meier analysis of OS with regard to European Society for Blood and Marrow Transplantation (EBMT) risk score [11] in a simplified adaption into three risk categories. Respective clinical data for risk score calculation was available for 163 patients. (**d**) Left panel: Inclusion of the risk factor *low HED^total^* to the EBMT risk score [11], yielding three major risk categories. Right panel: Kaplan–Meier analysis of OS in the AML patient cohort (*n* = 163) with regard to the adapted risk score. Abbreviations: HED indicates HLA evolutionary divergence; OS, overall survival; HSCT, hematopoietic stem cell transplantation; HLA-id., HLA-identical; mo, months; EBMT risk score, European Society for Blood and Marrow Transplantation risk score [11]; and ^#^, versus early stage.

Observing independent effects of low HED^class I^ and low HED^DR^ on outcome, we then analyzed the effect of HED^total^ (HED^class I + DR^) on survival. To define a cutoff value for HED^total^, the Youden index was determined based on a receiver operating characteristic curve analysis and the optimal cutoff value implemented thereafter (HED^total^ < 13.47, corresponding closely to the 25th percentile of HED^total^ = 13.20). Strikingly, low HED^total^ as defined by our cutoff value showed the strongest association with adverse OS in our AML patient cohort (HR for death 2.2, 95% CI 1.2–3.1, Figure 1b). The 5 year OS after allogeneic HSCT was 64.9% in patients with high HED^total^ and 29.7% in patients with low HED^total^ (Figure 1e, *p* < 0.001). Notably, HED^total^ remained a significant prognostic factor for OS when excluding patients with homozygosity in one or more HLA class I or DR alleles (*n* = 126, HR for death 2.5, 95% CI 1.2–5.1, *p* = 0.02, Figure 2a). A subgroup analysis regarding donor type showed a stronger impact of HED^total^ in patients with matched unrelated donors when compared with patients with an HLA-identical sibling donor (Supplemental Figure S1a,b).

**Figure 3:**
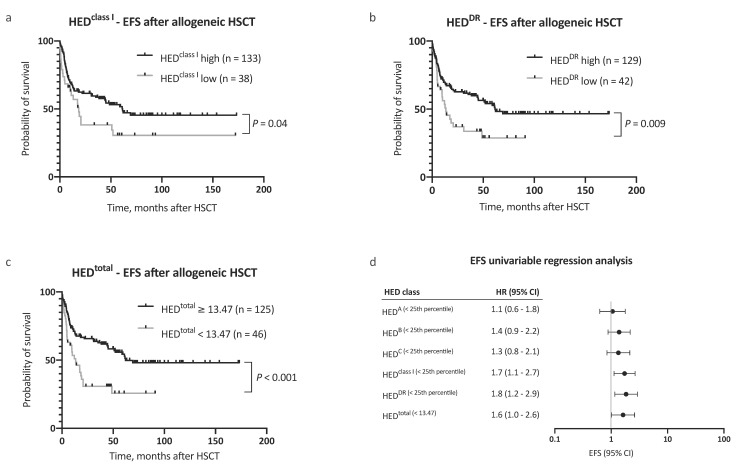
Analysis of event-free survival. (**a**–**c**) Kaplan–Meier analysis of event-free survival (EFS) with regard to (**a**) HED^class I^, (**b**) HED^DR^, and (**c**) HED^total^. (**d**) Univariable Cox regression analysis of the impact of low HED on EFS. Abbreviations: CI indicated confidence interval; EFS, event-free survival; HED, HLA evolutionary divergence; and HSCT, hematopoietic stem cell transplantation.

In a multivariable Cox regression analysis, HED^total^ was an independent, and the strongest single, risk factor for adverse OS, with consideration of other risk factors (as proposed by the EBMT score [11], HR 2.7, 95% CI 1.6–4.6, Figure 2b). Inclusion of HED^total^ in the risk assessment further allowed for a clear distinction between groups with regard to outcome (Figure 2c,d). In addition to OS, a negative impact on event-free survival (EFS) was noted for low HED^class I^, HED^DR^, and HED^total^ (Figure 3a–d). The 5 year EFS, after allogeneic HSCT, was 25.7% in patients with low HED^total^ and 54.4% in patients with high HED^total^ (Figure 3c, *p* < 0.001). HED^total^ was further associated with a higher risk (HR 2.2, 95% CI 1.3–3.6, *p* = 0.005) and cumulative incidence of relapse after allogeneic HSCT (Grey’s test *p* = 0.01, Supplemental Figure S1c). The median time to relapse was similar in patients with low and high HED^total^ (5.0 and 7.3 months, respectively).

Further supporting the significance of HLA divergence for GvL efficacy, HED^total^ was not associated with OS in patients undergoing HLA-mismatch or haploidentical allogeneic HSCT (*n* = 71, excluded from survival analysis, Supplemental Figure S1e), where GvL effects were limited to HLA allele matches. The overall frequency of graft-versus-host disease was similar in patients with low and high HED^total^ (Supplemental Figure S1d).

## 3. Discussion

In line with recent findings reported for immune checkpoint inhibitor therapy [7,16], we here show for the first time that low HED is strongly associated with adverse outcome in AML patients undergoing allogeneic HSCT. Our findings indicate that HLA divergence directly defines the ability to present diverse TAAs, and is thus a key variable for the success of GvL-mediated anti-tumor responses.

Effective T cell cancer immune control requires functional tumor-specific T cells as well as adequate presentation of TAAs via HLA molecules [17,18,19]. As each HLA allele presents a distinct peptide repertoire, a high HLA divergence allows for the presentation of a broader spectrum of HLA peptide binders, including TAAs [4,6,7,8,20]. Low HED in turn confines antigen presentation to a narrow spectrum of peptide binders and restricts anti-tumor immune responses to fewer T cell epitopes. Consistently, allele-specific loss of HLA antigen expression was recently described as an immune escape mechanism [21,22,23]. This might be of particular significance in low-mutational burden malignancies, including AML, where the spectrum of TAAs is additionally confined by a paucity of mutated neoantigens [23,24,25].

The immunological benefit of high HED, and thus immunopeptidome diversity, was first described in infectious diseases [26,27,28], but its relevance for antigen presentation, enabling more effective recognition of altered cells, has recently also been reported in cancer immunology [7,16]. Our findings indicate that HED is also a key determinant for GvL effects after allogeneic HSCT, a prime example of T-cell-based immunotherapy. The observed strong association of low HED with a higher relapse rate, and adverse survival, thereby directly reflects impaired immunological disease control, when TAA presentation is confined by low HED.

Remarkably, both HED of HLA class I and HLA-DR antigens were strong independent determinants of survival, highlighting the significance of both CD8^+^ and CD4^+^ T cells for GvL immune responses [29,30,31,32]. The particularly strong association of HED^DR^ with survival thereby supports the increasingly appreciated role of CD4^+^ T cells in anti-tumor immune responses. While the contribution of HLA class II antigen presentation and CD4^+^ T cells to GvL effects remains vaguely defined, our findings support their described pivotal role for anti-tumor immunity, as CD4^+^ T cells exert multiple direct anti-tumor effects and orchestrate CD8^+^ T cell responses at the same time [29,31,32,33,34].

Subgroup analyses in our study provide additional noteworthy findings: The association of HED with survival in fully heterozygous patients suggests that divergence contributes to immunopeptidome abundance, independently of heterozygosity, thus supporting both the heterozygote and divergent advantage hypothesis first formulated in infection immunology [4,16,35]. Furthermore, the more distinct impact of HED in patients with matched unrelated donors is in line with reports suggesting that HLA-DP and HLA-DQ antigens also contribute to TAA presentation [23,32,36,37]. As sibling donors are more likely to have matching HLA-DP and HLA-DQ alleles, TAA presentation via these antigens might partially compensate for low divergence of HLA class I and HLA-DR alleles. It will be of interest to evaluate the divergence of these not routinely assessed HLA class II antigens in future studies.

The less distinctive effect of HED on EFS, when compared to OS, further indicates that HLA divergence might also be of immunological significance for relapse therapies in AML, including donor lymphocyte infusions and treatment with hypomethylating agents [38,39,40].

Non-relapse mortality and morbidity after allogeneic HSCT call for a risk-stratified treatment approach, but predicting outcome of AML patients after allogeneic HSCT remains difficult [41]. Novel predictive markers are therefore needed, and HED, due to its strong association with survival, should thus be considered in patients evaluated for allogeneic HSCT. As the natural limitations of retrospective analyses apply to our study, analysis of an independent, ideally prospective, validation sample would be helpful to validate the optimal HED cutoff value for clinical use.

## 4. Materials and Methods

To determine the impact of HED on survival after allogeneic HSCT, we retrospectively analyzed the outcome of 242 AML patients who underwent allogeneic HSCT at the University Hospital Tübingen, Germany, a tertiary hematology–oncology and hematopoietic stem cell transplantation referral center from 2005 to 2019. Complete patient characteristics are provided in Table 1. All patients (*n* = 242) contributed to the calculation of HED quartiles, while only patients undergoing HLA-matched allogeneic HSCT (*n* = 171) were included in the survival analysis. HLA typing was carried out by the Department of Hematology and Oncology, University Hospital Tübingen, Germany.

HED was calculated as pairwise differences implementing a Grantham distance metric [10], using a Perl script, available online (https://granthamdist.sourceforge.io/), as previously described [4]. The respective protein sequences of the peptide binding domain (exon 2 and 3 for HLA class I, exon 2 for HLA-DR) were obtained from the international immunogenetics project’s HLA database [42]; exon annotation was performed with Ensembl [43]. HED was calculated for HLA-A, -B, -C, and -DR alleles, and low HED was defined as HED below the 25th percentile, while high HED was defined as HED equal to or above the 25th percentile. As they are not routinely assessed, HLA-DP and HLA-DQ antigens were not considered.

GraphPad Prism 8 (GraphPad Software, La Jolla, CA, USA), SPSS 26 (IBM, Armonk, NY, USA), and R 4.0.2 (R Foundation for Statistical Computing, Vienna, Austria) were used for statistical analysis. Event-free survival (EFS) and overall survival (OS) were defined as time from allogeneic HSCT to relapse or death, and death from any cause, respectively. If no event occurred, data were censored at the last recorded patient contact. The log-rank test was used for the comparison of Kaplan–Meier estimates between different groups of patients with a significance level of α = 0.05. The median follow-up time was assessed using a reverse Kaplan–Meier estimate. The Cox proportional hazards regression model was used to assess the effect of multiple variables on EFS and OS. Cumulative relapse incidence was calculated treating non-relapse mortality as a competing risk. All *p*-values are two-sided. The study was performed according to the guidelines of the local ethics committee (406/2019BO2) and in accordance with the Declaration of Helsinki protocol.

## 5. Conclusions

Our study strengthens the role of HED as a fundamental and defining metric of immunopeptidome diversity. As a surrogate for the ability to present diverse TAAs, HED is critical for T cell cancer immunity and a key determinant of the success of T-cell-based immunotherapy approaches. In patients evaluated for allogeneic HSCT, HED is an easily accessible prognostic marker with high impact on survival, and should be considered during risk assessment.

## Figures and Tables

**Table 1:** Patient characteristics.

Characteristic	All Patients (*n* = 171)	HED^total^ Low (*n* = 46)	HED^total^ High (*n* = 125)	*p-Value*
Follow-up after HSCT (mo.)				
*Median*	59.4	51.6	62.6	
*Range*	2.1–173.3	2.1–173.3	5.5–127.8	
Age at Allo-HSCT (yr.)				
*Median*	56	59	55	0.30 ^†^
*Range*	21–75	21–71	22–75	
Sex (no. (%))				
*Male*	98 (57.3)	28 (60.9)	70 (56.0)	0.57 ^‡^
*Female*	73 (42.7)	18 (39.1)	55 (44.0)	
Karnofsky index (no. (%))				0.23 ^§^
*≥90*	115 (67.3)	31 (67.4)	84 (67.2)	
*<90*	35 (20.5)	12 (26.1)	23 (18.4)	
*n.a.*	21 (12.3)	3 (6.5)	18 (14.4)	
WHO 2016 subtype (no. (%))				
*RGN*	86 (50.3)	21 (45.7)	65 (52.0)	0.22 ^§^
*MDS-related*	31 (18.1)	13 (28.3)	18 (14.4)	
*Therapy-related*	8 (4.7)	2 (4.3)	6 (4.8)	
*NOS*	46 (26.9)	10 (21.7)	36 (28.8)	
EBMT risk score (no. (%))				
*1*	3 (1.8)	1 (2.2)	2 (1.6)	0.23 ^§^
*2*	28 (16.4)	6 (13.0)	22 (17.6)	
*3*	52 (30.4)	8 (17.4)	44 (35.2)	
*4*	32 (18.7)	11 (23.9)	21 (16.8)	
*5*	38 (22.2)	14 (30.4)	24 (19.2)	
*6–7*	10 (5.8)	3 (6.5)	7 (5.6)	
*n.a.*	8 (4.7)	3 (6.5)	5 (4.0)	
ELN risk group ^¶^ (no. (%))				
*Favorable*	19 (11.1)	5 (10.9)	14 (11.2)	0.81 ^§^
*Intermediate*	110 (64.3)	32 (69.6)	78 (62.4)	
*Adverse*	26 (15.2)	9 (19.6)	17 (13.6)	
*n.a.*	16 (9.4)	0 (0.0)	16 (12.8)	
HSCT setting (no. (%))				
*1*st *line consolidation*	106 (62.0)	25 (54.3)	81 (64.8)	0.44 ^§^
*Salvage therapy ^||^*	42 (24.6)	14 (30.4)	28 (22.4)	
*Relapse*	23 (13.5)	7 (15.2)	16 (12.8)	
Conditioning regimen (no. (%))				
*FLAMSA*	10 (5.8)	4 (8.7)	6 (4.8)	0.32 ^§^
*FLAMSA-Flu/Bu*	41 (24.0)	16 (34.8)	25 (20.0)	
*Flu/TBI*	16 (9.4)	4 (8.7)	12 (9.6)	
*Cy/TBI*	19 (11.1)	3 (6.5)	16 (12.8)	
*Bu/Cy*	24 (14.0)	5 (10.9)	19 (15.2)	
*Flu/BCNU/Mel*	8 (4.7)	1 (2.2)	7 (5.6)	
*Flu/Bu*	27 (15.8)	8 (17.4)	19 (15.2)	
*Flu/Treosulfan*	18 (10.5)	2 (4.3)	16 (12.8)	
*Other*	8 (4.7)	3 (6.5)	5 (4.0)	
Remission at HSCT (no. (%))				0.16 ^§^
*CR/CRi*	103 (60.2)	22 (47.8)	81 (64.8)	
*PR*	23 (13.5)	8 (17.4)	15 (12.0)	
*RD*	37 (21.6)	13 (28.3)	24 (19.2)	
*n.a.*	8 (4.7)	3 (6.5)	5 (4.0)	
Donor (no. (%))				
*HLA-ident sibling*	63 (36.8)	23 (50.0)	40 (32.0)	0.03 ^§^
*HLA-ident foreign donor*	108 (63.2)	23 (50.0)	85 (68.0)	

Abbreviations: BCNU, Carmustine; Bu, Busulfan; CR/PR, complete/partial remission; Cy, Cyclophosphamide; ELN, European Leukemia Network; FLAMSA, fludarabine cytarabine, amsacrine ± irradiation (depending on additional therapy); Flu, fludarabine; HCST, hematopoietic stem cell transplantation; Mel, melphalan; mo., months; n.a., not available; NOS, not otherwise specified; RD, refractory disease; RGN, recurrent genetic alterations; yr., years; TBI, total body irradiation (combined ≥ 8 Gy); ^†^, Mann–Whitney-Test; ^‡^, Fisher’s exact test; §, Pearson’s chi-squared test; ^¶^, according to the respective current ELN classification [14,15] (when applying the ELN 2010 classification [15], *Intermediate-I* and *Intermediate-II* were grouped together); and ^||^, < CR after induction therapy.

## Supplementary Materials

The following are available online at https://www.mdpi.com/2072-6694/12/7/1835/s1, Figure S1: Subgroup analyses, cumulative relapse incidence and GvH frequency.
