# Supplementary material for: HLA Evolutionary Divergence as a Prognostic Marker for AML Patients Undergoing Allogeneic Stem Cell Transplantation
Source: Cancers (Basel). 2020 Jul 8;12(7):1835. doi: 10.3390/cancers12071835 (PMC7408841; doi:10.3390/cancers12071835)
Supplement: Supplementary file 1 [file cancers-12-01835-s001.pdf]

cancers

MDPI

Supplementary Materials

HLA Evolutionary Divergence as a Prognostic Marker for AML Patients Undergoing Allogeneic Stem Cell Transplantation

Malte Roerden, Annika Nelde, Jonas S. Heitmann, Reinhild Klein, Hans-Georg Rammensee, Wolfgang A. Bethge and Juliane S. Walz

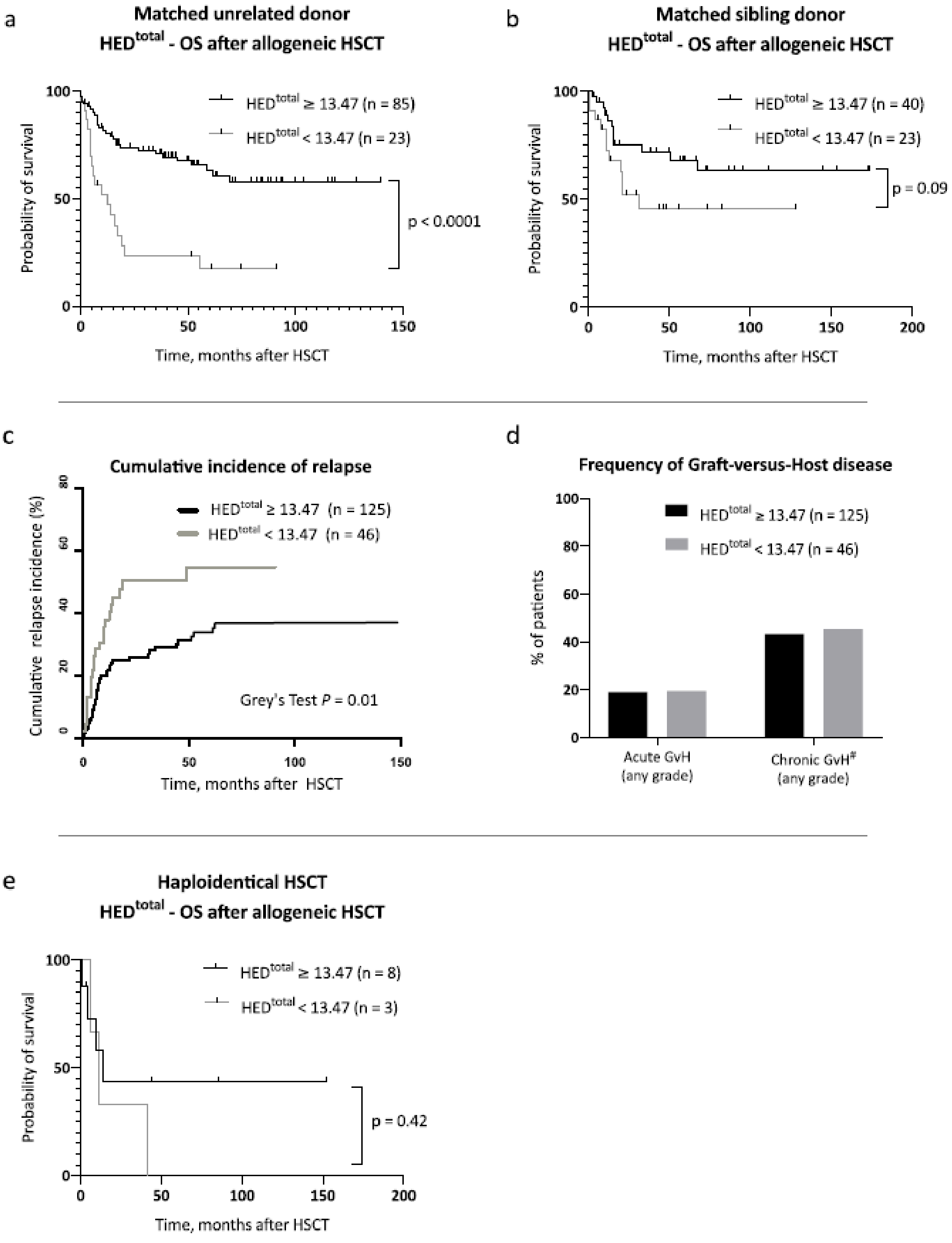

Figure S1. Subgroup analyses, cumulative relapse incidence and GvH frequency. (**a**,**b**) Kaplan-Meier analysis of OS in subgroups with regard to donor type. (**c**) Analysis of cumulative incidence of relapse (treating non-relapse mortality as a competing risk). (**d**) Overall frequency of acute and chronic GvH

depending on HED^{total}. (**e**) Kaplan-Meier analysis of OS for patients undergoing haploidentical HSCT. Abbreviations: GvH indicates Graft-versus-Host disease; HED, HLA evolutionary divergence; HSCT, hematopoietic stem cell transplantation; OS, overall survival; and #, only considering patients with OS > 3 months.

© 2020 by the authors. Licensee MDPI, Basel, Switzerland. This article is an open access article distributed under the terms and conditions of the Creative Commons Attribution (CC BY) license (http://creativecommons.org/licenses/by/4.0/).
